# Transcriptomic Analysis of Starch Biosynthesis in the Developing Grain of Hexaploid Wheat

**DOI:** 10.1155/2009/407426

**Published:** 2010-03-08

**Authors:** Boryana S. Stamova, Debbie Laudencia-Chingcuanco, Diane M. Beckles

**Affiliations:** ^1^Genomics and Gene Discovery Unit, USDA-ARS WRRC, 800 Buchanan Street, Albany, CA 94710, USA; ^2^Department of Plant Sciences MS3, University of California-Davis, 1 Shields Avenue, Davis, CA 95618, USA; ^3^Department of Neurology, School of Medicine, M.I.N.D Institute, University of California Medical Center, 2805 50th Street, Sacramento, CA 95817, USA

## Abstract

The expression of genes involved in starch synthesis in wheat was analyzed together with the accumulation profiles of soluble sugars, starch, protein, and starch granule distribution in developing caryopses obtained from the same biological materials used for profiling of gene expression using DNA microarrays. Multiple expression patterns were detected for the different starch biosynthetic gene isoforms, suggesting their relative importance through caryopsis development. Members of the ADP-glucose pyrophosphorylase, starch synthase, starch branching enzyme, and sucrose synthase gene families showed different expression profiles; expression of some members of these gene families coincided with a period of high accumulation of starch while others did not. A biphasic pattern was observed in the rates of starch and protein accumulation which paralleled changes in global gene expression. Metabolic and regulatory genes that show a pattern of expression similar to starch accumulation and granule size distribution were identified, suggesting their coinvolvement in these biological processes.

## 1. Introduction

Seed starch is the major storage compound in cereals providing as much as 80% of the calories consumed by mankind. This starch is also a major source of feed, fiber, biofuels, and biopolymers in many industrial applications. Understanding the molecular basis of starch physicochemical properties and the control of its synthesis in the seed is a necessary step in improving and modifying starch properties tailored to an increasing variety of end-uses.

Starch is deposited as discrete, water-insoluble semicrystalline granules in the plastid. It is composed of two glucose polymers, called amylose and amylopectin, which share the same basic glucan structure but differ in length and degree of branching. Amylose is essentially a linear molecule of *α*-1,4-linked glucose residues with a few *α*-1,6-glycosidic linkages. The degree of polymerization of glucose in amylose molecules is species dependent and averages about 800 residues in wheat [1]. Amylopectin molecules, on the other hand, are much larger (up to millions of residues) and highly branched with a high frequency of *α*-1,6-glycosidic linkages [2]. The branching of the glucan chains of amylopectin occurs with regular periodicity [3] and its length and pattern are critical for the proper formation of the starch granule. 

In wheat, starch granules exhibit a bimodal size distribution—a characteristic unique to members of the grass Triticeae family. The starch granules, designated A-, and B-starch granules [4], can be distinguished based on size, shape, relative proportion, and the timing of their initiation in the endosperm—a process which, presumably, is under a defined genetic program. A-granules are lens-shaped, 10–50 *μ*m in diameter, and make up to 70% of the volume and 10% of the total number of starch granules [5, 6]. In contrast, B-granules are spherical, 5–9 *μ*m in diameter, and represent ~30% of the volume and 90% of the total number of granules. More recent evidence indicates the presence of C-type starch granules with diameter less than 5 *μ*m [4, 7]. The small size of the C-granules makes them difficult to isolate and quantify which commonly leads to their being classified with B-granules. A-granules are formed around 4–14 days postanthesis (DPA) when the endosperm is still actively dividing [4, 8, 9]. B-granules are initiated at about 10–16 DPA in stromules (stroma containing tubules) that are extruded from A-granule containing plastids [6, 7], and the small C-granules first appear about 21 DPA [7]. The genetic basis of the multimodal size distribution of starch in wheat and barley is of great interest because the physiochemical properties of each type of starch granule vary and contribute to the food and industrial end-uses of Triticeae starch [10–12]. 

The posttranslational control of many enzymes involved in starch biosynthesis has been well documented [13–15]. In contrast, the transcriptional regulation of the genes coding for these enzymes has not yet been fully explored. Transcriptional regulation may be a more important mechanism for long-term control of genes expression especially during caryopsis development. Several studies show that carbohydrate synthesis genes are strongly regulated by sugars—especially sucrose [16] and glucose [17]—and that sugars are important sensors and regulators of multiple pathways [16, 18–20]. 

In this report, results of a global gene expression profiling experiment were overlaid with the analysis of soluble sugar accumulation, starch content, and starch granule particle size distribution on the same batch of biological materials used for the microarray experiment. Results showed multiple temporal expression patterns of key genes involved in starch synthesis, suggesting the relative importance of the different enzymes throughout caryopsis development. Correlative analysis identified genes that showed similar patterns of expression to the accumulation profiles of starch and amylose and the distribution of starch granules—suggesting these as possible candidate genes for further investigation for their roles in seed starch synthesis and potential targets for modulating carbohydrate metabolism in wheat by genetic engineering or molecular breeding efforts.

## 2. Materials and Methods

### 2.1. Plant Material

*Triticum aestivum* L. cv. Bobwhite and cv. Hereward plants used for tissue sampling and RNA extraction for cDNA and oligoarrays experiments, respectively, were grown in the greenhouse under conditions as previously described [21, 22]. Caryopses from the head of the main stem of each Bobwhite plant were harvested at different time-points for fresh-weight, dry-weight, total starch, and total protein content determinations.

### 2.2. Starch, Protein, Sugar, and Amylose Content

The total starch and total protein data obtained from our earlier published work [21] were used in this paper. Briefly, wheat caryopses from 10 to 25 heads per time-point were independently harvested, freeze-dried, and ground to flour using the UDY mill (UDY Corporation, Fort Collins, CO, USA) for measurement of total starch and protein. The Megazyme total starch determination assay kit (Megazyme International, County Wicklow, Ireland) was used for starch determination. Amylose was determined for caryopses at 7, 10, 14, 18, 21, 25, 28, 35, and 50 DPA using the Megazyme amylose/amylopectin determination kit. Total protein content was determined by N combustion analysis with a FlashEA 1112 N/Protein analyzer (ThermoFisher Scientific, Waltham, MA USA). 

Soluble sugars were determined at 7, 14, 21, 28, and 35 DPA by boiling each sample in 5 mL 80% (v/v) ethanol for 5 minutes. The samples were centrifuged at 4000 × g for 15 minutes, the ethanol was decanted, and an additional 5 mL ethanol added to the pellet which was resuspended and boiled again for 5 minutes. This was done three times, each time pooling the ethanol soluble fraction. The ethanol was removed by drying samples in a speed-vac and the residue reconstituted in 300 *μ*L of water. Samples were filtered through a 0.45 *μ*m filter and injected onto a Hamilton RX-10 Anion exchange column (Hamilton Company, Reno, NV). 

Sucrose, fructose, and glucose were measured by HPLC on a Dionex BioLC system (Dionex, Sunnyvale, CA) with Pulse Amperometric Detection. The gradient elution schedule consisted of 15 minutes of 15% of 200 mM NaOH, then 30% over 7 minutes, and 15% NaOH for 10 minutes. Standard solutions of sucrose, fructose, and glucose (Fluka BioChemika Company; Steinheim, Germany) were mixed and 10 *μ*L injected on to the column at a flow rate of 2.0 mL/min. Trehalose eluted at 2.7 minutes, glucose at 6.1 minutes, fructose at 7.6 minutes, and sucrose at 9.6 minutes. The amounts were expressed as milligrams per gram of dry weight of tissue.

### 2.3. Starch Granule Distribution Determination

Starch granules from caryopses at each time-point were isolated and purified using a established protocol [23]. Freeze dried whole caryopses were ground in a mortar and pestle and homogenized gently in 0.5 M NaCl. The homogenate was filtered with a 90 *μ*M mesh filter, and the retentate was collected and gently ground further to release starch. This procedure was repeated until most of the starch was washed off from this fraction. No preferential loss of small-granules was detected as monitored by iodine-staining of the flow-thru in each wash. Starch fractions collected were resuspended by vortexing in 5 volumes of 0.5 M NaCl, and then centrifuged at 10,000 g for 10 minutes. Debris at the starch-liquid interface was carefully removed and the pellet resuspended in 0.5 M NaCl and then recentrifuged. This step was repeated until most of the debris was removed. The pellet was then washed in water (three times), 2% (v/v) SDS (twice), in water (three times), and once with 80% (v/v) acetone and then dried overnight. The fractionated starch was checked for debris by light microscopy. Approximately 50 mg of purified starch was diluted in 50 mL of water and particle size analysis was processed using the Horiba 900 Laser Scattering Particle Size Distribution Analyzer (Irvine, CA). For the 7 DPA sample, 13 mg of purified starch was analyzed. For granule volume calculations the correction factor developed by Wilson et al. [4] was adapted wherein all granules ≤5 *μ*m were considered spherical and those bigger than 5 *μ*m in diameter were considered oblate spheroid with thickness of 5 *μ*m and varying equatorial diameters.

### 2.4. Starch Granule Scanning Electron Microscopy

Starch granules purified from the different developmental stages were dusted on the surface of a carbon adhesive tab and sputter coated with gold palladium particles using Dentum Vacuum Desk II. Samples were viewed at 2.0 kV with the Hitachi Model S-4700 scanning electron microscope.

### 2.5. Gene Expression Analysis with DNA Microarrays

Two sets of microarray data were examined for expression of genes involved in starch metabolism in the developing wheat caryopsis. The first data set was obtained from our previous work [21] which utilized an 8 K cDNA array enriched for genes expressed in the endosperm. The expression of genes in developing caryopses of spring wheat *T. aestivum* cv. Bobwhite was examined using RNA from six time-points (3, 7, 14, 21, 28, and 35 DPA) which covered the critical stages in caryopsis development—from coenocytic to desiccation stage. The 3 DPA data were omitted for correlative analyses since starch accumulation begins at about 5 DPA and at earlier times nonendosperm tissues predominate. Data visualization and coordinate transcript expression analyses were accomplished using the built-in statistical modules in Genespring GX software (Agilent Technologies, Santa Clara, CA). The TIGR website (http://compbio.dfci.harvard.edu/tgi/) was used to determine if different ESTs belonged to the same contig, therefore belonging to the same tentatively unique gene.

The second data set was derived from a time-series experiment in grain development [22] using the Affymetrix Wheat Genome short oligo DNA expression arrays (referred to hereafter as the oligoarray) with 61,127 probes representing 55,052 potential genes. In this experiment, the expression of genes was examined in the developing caryopses of winter wheat *T. aestivum* cv. Hereward caryopses at ten time-points (6, 8, 10, 12, 14, 17, 21, 28, 35, and 42 DPA) covering the onset of grain-filling stage to grain maturation. New analyses were carried out on 20 oligoarrays corresponding to the caryopses developmental series downloaded from the Gene Expression Omnibus (E_MEXP-1193, http://www.ncbi.nlm.nih.gov/geo/) to allow extraction of gene expression profiles relevant to the current report. Probe set signal normalization and summarization was carried out using GCRMA [24], the modified Robust Multiarray normalization algorithm that takes into consideration the GC content of each probe, as implemented in Genespring software. A list of differentially expressed genes was generated using one-way ANOVA using SAS version 9.0. The wheat oligoarray database at http://www.plexdb.org/ [25] was used to verify the most recent annotations for the probesets. The NCBI EST assembly nomenclature was used to name the oligoarray probes; that is, Ta.6869 is *T. aestivum* unigene 6869.

For the purpose of this work, a probe set is deemed to represent a potentially unique wheat gene and the accumulation of its transcript as measured by the signal intensities in each probe set represents the “expression” of the gene. In this study, it will be understood that “transcript accumulation” and “gene expression” will refer to gene transcript steady-state levels and will be considered to approximate the level of expression of the relevant gene. The MapMan software [26] was adapted to display the change in transcript expression during wheat caryopsis development using a color code, which were overlaid onto a custom-made sucrose-to-starch pathway.

## 3. Results and Discussion

### 3.1. Carbohydrate Composition in Developing Wheat Caryopsis and Correlated Gene Expression

#### 3.1.1. Soluble Carbohydrates

**Figure 1:**
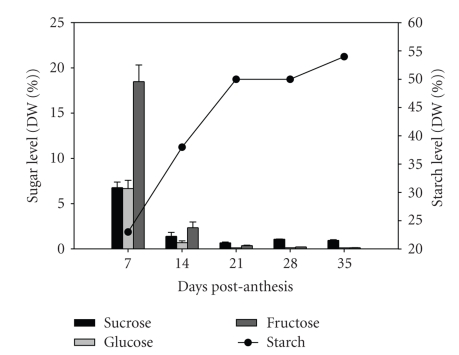
*Soluble sugar content during caryopsis development. *Starch and sugar content of the developing wheat caryopsis. Starch and sugar were measured by HPLC in dried whole caryopses; the same batch of biomaterial used for the microarray analysis. Values represent the percentage of each sugar in caryopsis tissue expressed as a percent dry weight (% DWT) of caryopsis tissue. Values are the mean ± Standard Error of the Mean (SEM) of 3-4 independent measurements of 3 biological replicates per time-point. The starch data are the mean of 2–5 biological replicates per time-point.

The soluble sugar content measured in developing caryopses showed that sucrose, fructose and glucose were at their highest levels at 7 DPA, then decreased to lower levels by about 21 DPA, and remained fairly constant through 35 DPA (Figure 1). Fructose was the predominant sugar at 7 DPA caryopses and was about 2.5 times higher than sucrose and glucose, and sucrose has lower levels than fructose at 7 and 14 DPA. Sucrose levels became the most abundant sugar by 21 DPA with the relative ratio of sucrose to both glucose, and fructose rising through 35 DPA, which may indicate its active import into the endosperm. Measurements of sucrose, glucose and fructose contents were in agreement with an earlier published work [27] in desiccated whole kernel flours from hard red spring and durum varieties. 

There were 49, 377, and 409 genes which expression correlated with sucrose, glucose, and fructose pattern of accumulation, respectively (see Supplementary Material 1 available online at doi:10.1155/2009/407426). Among the specific genes correlating with changes in sucrose levels include a putative transcription factor (TF) described as BTF3, as well as with another putative TF, some carbohydrate-metabolic genes, and an ATP/ADP carrier protein (Table 1). Glucose accumulation was tightly linked with the transcript accumulation profile of calmodulin, two putative TFs, a 14-3-3 protein, a MADS-box protein 9, a putative PGI, putative and probable protein kinases as well as other carbohydrate-metabolic genes (Table 1 and Supplementary Material 1). The fructose accumulation profile shows very high similarity to the pattern of expression of a CDPK protein-like calmodulin gene from rice that may play a role in signal transduction pathways that involve calcium as a second messenger as well as other regulatory and carbohydrate-metabolism genes (Table 1). 

When the data were further examined for genes correlating to more than one of the 3 sugars, only two showed shared genes in common, both involving glucose. Of the 23 genes that correlated with glucose and sucrose, only the ATP/ADP carrier protein was known to be involved in the starch synthesis pathway; there were no regulatory factors identified (see Supplementary Material 2). Of the 249 genes that correlated well with the changes in the levels of both fructose and glucose were metabolic and transcriptional regulatory genes, for example, putative pyruvate kinase, a yabby protein, a probable kinase, 14-3-3 regulatory proteins, a PISTILLATA-like MADS-box protein, and other TFs. 

We observed 249 overlapping coexpressed genes with the levels of fructose and glucose in the grain, even though fructose levels were about three times higher than glucose levels at 7 and 14 DPA (Figure 1). There were 23 genes coordinately expressed with both sucrose and glucose levels while there were none for sucrose and fructose. It is difficult to explain the high correlation between transcripts that have similar accumulation profile as both glucose and fructose levels and the difference in the number of transcripts that follow sucrose/glucose (23) versus sucrose/fructose levels (0). What our results perhaps signify is that the metabolism of glucose, rather than fructose, more closely reflects changes in sucrose and starch metabolism. This is consistent with starch as the major sink for sucrose, and through its activated form ADP-glucose, glucose is the precursor for starch biosynthesis. However this is not reconciled with the report that glucose and fructose appear to contribute equally to starch metabolism [28]. There are very few studies that show the extent to which fructose is a regulator of global gene expression in cereal endosperm or any other plant organ, and the interrelationship of sugars to starch formation requires more research [29].

#### 3.1.2. Starch, Amylose, and Amylopectin

**Figure 2:**
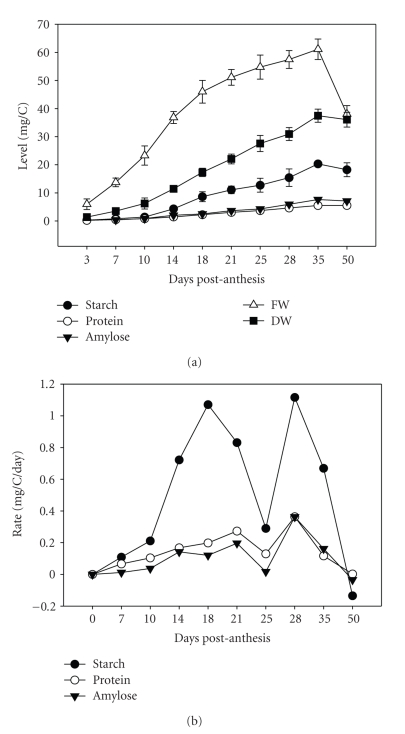
*Amount and rate of storage product accumulation in the developing wheat grain. *For both starch and protein assays, three determinations were made per biological sample, and 2–5 samples were used per time-point. The amylose data is the mean of the measurements of 3-4 biological replicates per time-point. (a) Level of starch, protein, amylose, fresh weight (FW) and dry weight (DW) per caryopis (mg/C) where C denotes caryopsis. (b) Rate of starch, protein and amylose accumulation (mg/C/day). Rate of accumulation was determined for each time interval.

The accumulation of storage reserves in the developing caryopsis showed that total protein, total starch, and the amylose content (Figure 2(a)) steadily accumulated from 10 DPA to a peak at 35 DPA followed by a slight reduction at 50 DPA. The amount of starch that accumulates as amylose increased from 7 DPA to 35 DPA of development. The change in amylose content in the caryopsis correlated with changes in granule distribution during grain maturation (see below) and transcripts of major seed storage proteins including *α*- and *γ*-gliadins (Table 1 and Table 2). The rate at which amylose accumulated was highly similar to an ADPG transporter (*Bt1*), two sucrose synthase (SuSy, EC 2.4.1.13) clones, and several regulatory proteins. 

Changes in the proportion of starch as amylopectin (total starch minus amylose) correlated well with four clones for putative Zn-finger proteins, a putative kinase interacting protein, a putative LRK1 (receptor-type) protein kinase, a probable protein kinase, as well as with carbohydrate-metabolic genes (Table 1). When the rate of both amylose and amylopectin accumulation is expressed as mg/caryopsis/day, high correlative patterns with the expression of a number of storage proteins, as well as genes encoding two Zn-finger, Leucine-rich WD domain containing proteins was found (see Supplementary Material 1). 

Detailed inspection of the rate of storage product accumulation revealed a biphasic pattern (Figure 2(b)). The rate of total protein accumulation increased steadily from 10 DPA and reaching a maximum rate at 21 DPA, then dropped 2-fold at 25 DPA, and then increased 3-fold at 28 DPA before declining as the grain matures. Similarly, the rate of total starch accumulation peaked at 18 DPA, dropped 3-fold at 25 DPA, and then increased 4-fold before declining through desiccation. The rate of amylose accumulation increased from the onset of the grain filling stage, peaking at 14 DPA, then leveling off until 21 DPA before declining 10-fold at 25 DPA. In contrast, the rate of total starch accumulation continued to increase from 10 DPA to 18 DPA then gradually declined until 25 DPA. The rate of total starch and amylose accumulation then increased 4- and 18-fold, respectively, to 28 DPA before the major decline through the end of grain development. 

To verify whether the biphasic pattern of storage reserve rate of accumulation we observed is not exclusive to the biological material we used, a set of external data independently obtained from another cultivar (courtesy of Dr. Frances Du Pont, USDA-ARS; personal communication) were similarly analyzed for rates of starch accumulation. Measurements of storage starch accumulation in the control (untreated) sample used in determining the effect of high temperature in developing caryopsis of *T. aestivum* var. Butte 86 [30, 31] showed the same biphasic pattern in the rate of starch accumulation (see Supplementary Material 3) consistent with our observation.

The observed biphasic pattern in the rates of protein, amylose and starch accumulation (Figure 2(b)), coincides with the biphasic reprogramming of genes during caryopsis development. We previously showed that the number of genes that are differentially expressed only between two consecutive time-points peaked twice—early to mid-development (between 3 to 14 DPA) and in later stages (between 21 to 28 DPA) of the caryopsis development [21]. In a separate study with a different wheat germplasm, significant changes in transcript abundance during maximum grain filling at 12–21 and at desiccation/maturation at 28–42 days after anthesis [22] were also reported. When looking at wheat storage protein expression, Kawaura et al. [32] found two distinct expression patterns, even within a single gene family, with peaks at 10 and 20 DPA. Taken together, these reports suggest that this biphasic changes may be a fundamental biological phenomenon in wheat caryopsis development.

#### 3.1.3. Starch Granule Size Distribution

**Figure 3:**
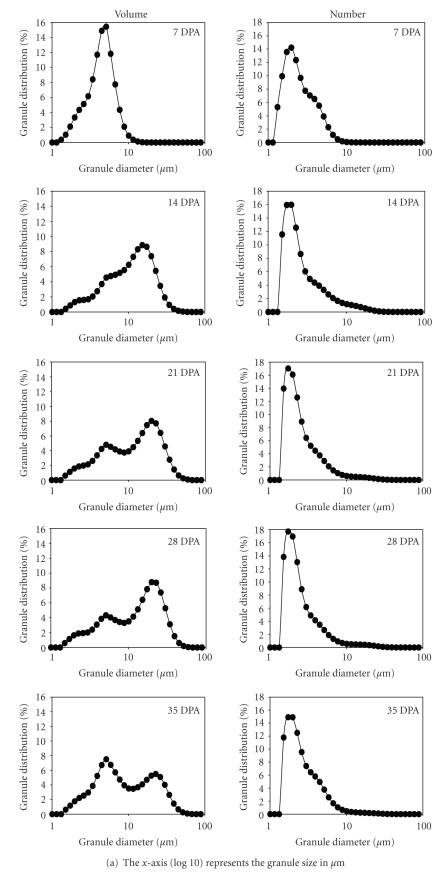

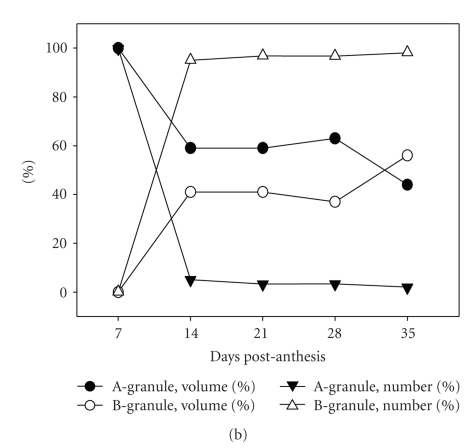
*Starch granule size distribution in developing grain.* (a) Column 1—granules as percent of total volume; column 2—as percent of total number. (b) Relative granule proportions in terms of their % number (num.) and % volume (vol.). Starch granules bigger than 10 *μ*m in diameter were considered A-granules and granules smaller or equal to 10 *μ*m in diameter were considered B-granules, except at 7 DPA when all granules were considered A-granules. For volume calculations all granules bigger than 5 *μ*m in diameter were considered oblate spheroid with thickness of 5 *μ*m and varying equatorial diameters.

**Figure 4:**
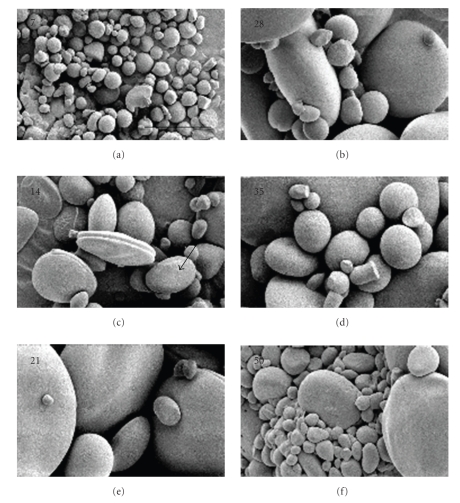
*SEM of developing starch granules.* Developing starch granules at different stages were viewed at the same magnification (4000X); the bar size represents 10 *μ*m. Starch granules at 7 DPA were 10 *μ*m or less in size. Arrow points to an oblate spheroid shaped A-granule at 14 DPA with less than 10 *μ*m in size. Starch granules with 5 *μ*m or less in size are present throughout the developing caryopsis.

The formation and size distribution of starch granules in developing caryopses were monitored by laser diffraction (Figure 3) and the morphology was examined by scanning electron microscopy (Figure 4). The data are consistent with the presence of a small granule fraction detected at 14 DPA, which are the B-type granules, and as previously reported granules detected at 7 DPA are of the A-type [7]. It is reasonable that some A-type granules that are still growing and are still <10 *μ*m in size may contribute to total volume of B-type granules at 14 DPA. In fact, SEM image of starch granules at 14 DPA shows the presence of oblate spheroid granules, a characteristic shape of growing A-type granules, at 10 *μ*m in diameter or less (Figure 4). At 21–28 DPA, the A-type granules continue to contribute most of the total starch granule volume (~58%) while the B-type granules make up most of the granule number (~37%) although the percentage is lower than at 14 DPA (~41%). This reduction could be due to the growth of A-type granules originally counted as B-granules at 14 DPA. We detected another increase in the number of granule <10 *μ*m at 28–35 DPA which may be due to the initiation of C-type granules. At 35 DPA the B- and C-type granules continued to contribute most of the total granule number (~98%). Growth of the C-granules probably increased its contribution to the volume of the small-sized granules from ~37% at 28 DPA to ~56% at 35 DPA. 

The rate of the change in volume of the A-granule population correlated with the expression profiles of two unknown genes (BE438268, *r* = 0.922; and BE422634, *r* = 0.832), a putative DEAD box RNA helicase (*r* = 0.849), and chitinase2 (*r* = 0.811). The profile of B-granule distribution (number and volume) and the ratio of B- to A-granules in terms of their volume and number correlated predominantly with the changes in transcriptional expression of storage protein genes (see Table 1, Table 2; and supplemental material 1). The distribution of the A-granules in terms of their percent volume correlated well with the expression profiles of two genes encoding regulatory proteins. In terms of their percent number, A-granules correlated with the expression profiles of genes encoding several transcription factors and other carbohydrate metabolism proteins (Table 1). These identified genes could serve as entry points to gain further insights into the molecular basis of multimodal size distribution of starch granules in wheat. 

### 3.2. Expression of Key Genes in Starch Biosynthesis

**Figure 5:**
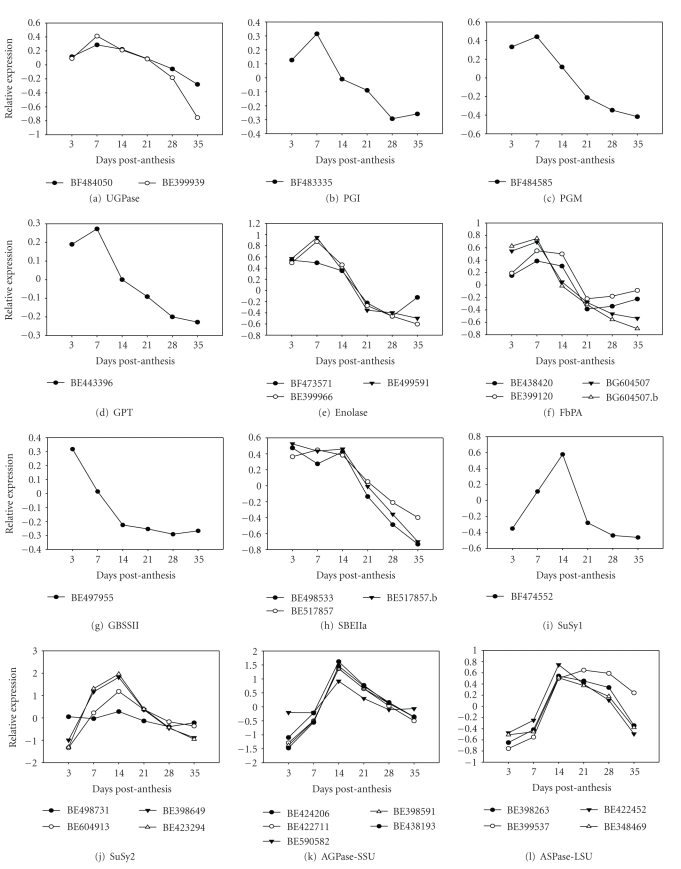

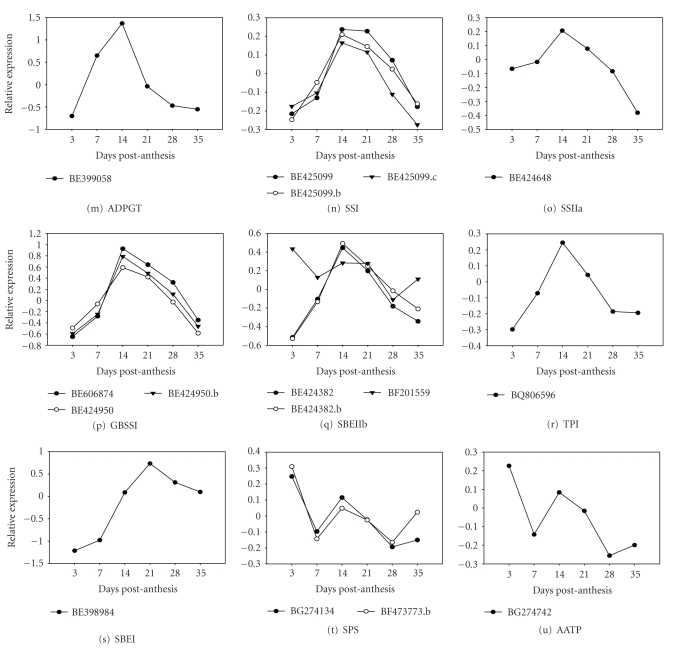
*Expression profile of genes involved in starch metabolism during caryopsis development organized by pattern of expression.* RNA samples used for the hybridization of the cDNA arrays were extracted from *T. aestivum* cv. Bobwhite caryopses. The expression profiles of genes were organized by pattern of expression: Early, Middle, or Late expresser. The *x*-axis shows the developmental stage time-points, and the *y*-axis represents the relative gene expression. Expression values are given as log 2-transformed normalized relative signal intensities, so that one unit on the *y*-axis represents an expression ratio of a factor of 2. GenBank accession numbers of the clones surveyed on the array are displayed on each chart.

**Figure 6:**
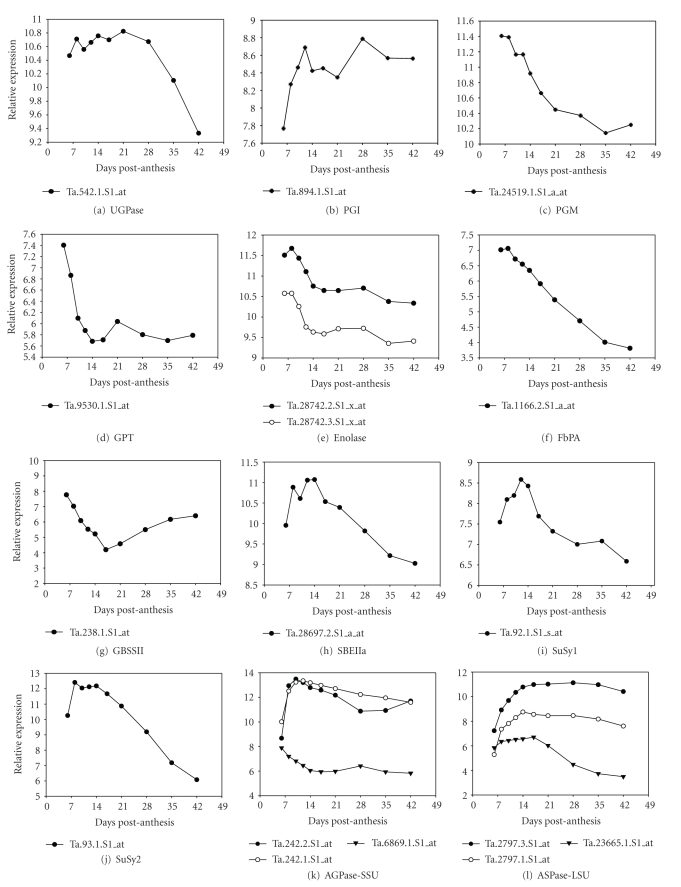

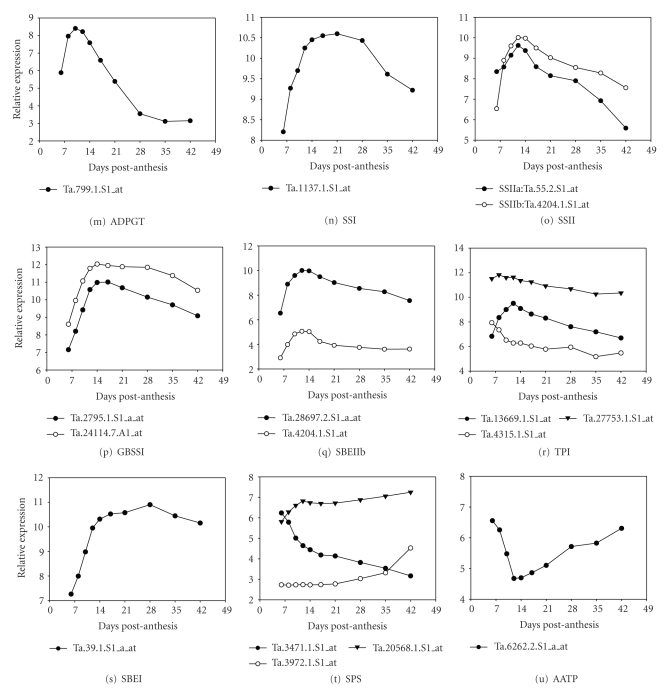
*Expression profiles of major starch biosynthetic genes using gene specific oligoprobes.* RNA samples used for hybridization of the oligoarrays were extracted from *T. aestivum* cv. Hereward caryopses. The *x*-axis shows the developmental stage time-points, the *y*-axis represents the relative gene expression (log 2). All the probesets are gene-specific based on Affymetrix annotation except for enolase and SuSy1, which could potentially cross-hybridize with other similar genes.

The expression profiles of genes known to be involved in the sucrose to starch biosynthetic pathway were examined; genes represented on the cDNA array and oligoarray are shown in Figures 5 and 6, respectively. The selected genes are presented according to their expression pattern as detected on the cDNA array that could be distinguished based on the age of the caryopsis at which transcript accumulation was maximal. The Early Expressers (Figures 5(a)–5(h)) had transcript levels highest at 3–7 DPA, when dry matter and storage reserves (starch and protein) start to accumulate, and then generally decline through caryopsis development. The Middle Expressers (Figures 5(i)–5(r)) had a bell-shaped pattern with a sharp increase and maximum level at about 14 DPA, when dry matter and storage reserves are rapidly accumulating, and then declining as the caryopsis matures. One gene has a profile (SBEI; Figure 5(s)) with bell-shaped pattern but peaks at 21 DPA and was categorized as a Late Expresser. Finally, two genes (Figures 5(t) and 5(u)) have similar Modulated patterns of expression throughout caryopsis development with lowest levels of transcripts at about 7 and 28 DPA. 

**Figure 7:**
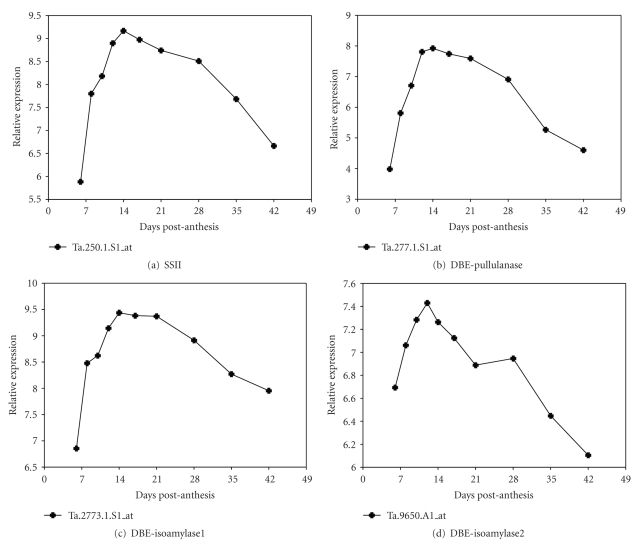
*Expression profile of other starch biosynthetic genes using the oligoarrays. *Other key starch biosynthetic genes present on the oligoarray but not the cDNA array platform.

Figure 7 shows the expression profiles of other critical genes represented on the oligoarray but not on the cDNA array. 

#### 3.2.1. ADP-Glucose Pyrophosphorylase

The first committed step to starch biosynthesis is catalyzed by ADP-glucose pyrophosphorylase (AGPase)—a tetrameric enzyme composed of two catalytic small subunits (SSUs) and two regulatory large subunits (LSUs), both encoded by several genes in wheat. The cDNA array detected expression of AGPase SSU (Figure 5(k)) and LSU (Figure 5(l)) genes both showed similar bell-shape patterns peaking around 14 DPA. AGPase SSU genes average expression changed about 8-fold, compared to 2.6-fold observed for LSU. For both SSU and LSU, in addition to multiple contigs showing the bell-shaped Middle Expresser pattern, a single sequence (BE590582 for SSU and BE399537 for LSU) showed less variation in expression. Both of these variants were sequences relatively 5′ in the coding regions of genes compared to the more 3′ sequences of the other contigs. The profile variation associated with more 5′ sequence is not known but may be related to the fact that target mRNA preparations tend to be 3′ biased. 

Examination of the AGPase SSU and LSU gene expression profiles using the oligoarray also detected different patterns of expression (Figures 6(k) and 6(l)). Two distinct expression patterns were observed for SSU. Ta.6869, which represents the plastidic isoforms (AY727927, [33]), showed little change in expression throughout development. In contrast, Ta.242 expression rose rapidly from 6 DPA to 8 DPA and remained high thereafter. Ta.242 target sequence shows about 99% sequence identity to a cytosolic SSU gene (EF405961) and 95% sequence identity to a plastidic gene (EU586278). It has been shown previously that the plastidic and cytosolic isoforms of SSU can be derived from the same gene by differential splicing [34] with the difference in sequence primarily confined to the 5′ end of the gene. Ta.242 also matches the set of AGPase SSU sequences on the cDNA array which suggests that they are encoded by the same gene.

Two patterns were also detected with the oligoarray for AGPase LSU gene expression. Ta.23665 showed a slight increase in expression from 6 DPA to 8 DPA and then gradually dropped from 17 DPA onward, whereas the two Ta.2797 probesets, derived from different regions of the same sequence, showed peaked expression at 14 DPA and remained high thereafter. Ta.23665 showed close similarity to a gene (GenBank ID X14348) that encodes the plastidic isoforms of the enzyme AGPase LSU. Ta.2797 matches all contigs from the cDNA array (Figure 5(l)), which presumptively are cytosolic isoforms.

#### 3.2.2. Starch Synthases

Starch synthases (SSs) catalyze the transfer of the glucose moiety from ADP-glucose to the reducing end of a preexisting *α*-1,4-linked glucan polymer. The starch synthases are grouped into five classes by DNA sequence; that is, SSI, SSII, SSIII, SSIV, and GBSS [35], and each has unique but occasionally overlapping roles. SSI (Figure 5(n)), SSIIa (Figure 5(o)), and GBSSI (Figure 5(p)) all showed bell-shaped expression profiles, which agreed with previous observations [30, 31]. GBSSII (Figure 5(g)) was an Early Expresser and its expression decreased through caryopsis development, consistent with its role in transient starch accumulation in the pericarp. The GBSSI contigs on the oligoarray (Figure 6(p)) were presumably two different homeologs, which the cDNA array failed to discriminate. 

The SSI, (Figure 6(n)), SSII (Figure 6(o)), and SSIII (Figure 7(a)) genes detected via oligoarrays also showed bell-shaped patterns of expression. The oligoarray discriminated between SSIIa (Figure 6(o), Ta.55) and SSIIb (Figure 6(o), Ta.4204) and also showed some variation in expression level. SSIIa expression was downregulated after 12 DPA at a faster rate compared to SSIIb. The general expression pattern of GBSSI (Figure 6(p), Ta.2795, and Ta.24114) was similar to that on the cDNA arrays (Figure 5(p)). Although the GBSSII expression patterns (Figures 5(g) and 6(g)) seem dissimilar, the cDNA array experiment included a high mRNA level at the 3 DPA point (Figure 5(g)) not included in the oligoarray.

#### 3.2.3. Starch Branching Enzymes

Starch branching enzymes (SBEs) catalyze the hydrolysis of an *α*-1,4-glucan linkage and form an *α*-1,6 glucosidic bond on C6 of a glucosyl moiety of an *α*-1,4 glucan to form a branch. The cDNA array expression profiles of SBEIIa (Figure 5(h)), SBEIIb (Figure 5(q)), and SBEI (Figure 5(s)) categorize these genes as Early, Middle, and Middle-Late Expressers, respectively. This result agrees with previous observations [36, 37], and points to different mechanisms for the temporal expression of the SBEs. In members of the Triticeae, a form of SBEI, termed SBEIc, is present exclusively in A-starch granules [38]. It contains two SBEI-like domains and is proposed to have originated by trans-splicing of a SBEI-like mRNA and a SBEI transcript. It is not possible to determine whether the SBEI clone on the cDNA array is SBEIc or SBEI because the SBEI-like domain sequence is present in both genes [39]. One clone for SBEI was present on the oligoarray (Figure 6(s), Ta.39), which showed a rapid increase from 6 DPA with a plateau from 14 to 42 DPA.

The three clones for SBEIIa on the cDNA array (Figure 5(h)) showed similar expression profiles with high transcript levels from 3 to 14 DPA and then gradually declined. There were three clones for SBEIIb on the cDNA array which are two different expression profiles (Figure 5(q)). BE424382 and BE424382.b, which showed a similar expression profile, are duplicates of the same clone—further confirming array data reproducibility. It should be noted that the BF201559 sequence aligns with the 5′-end of the *T. aestivum* SBEIIb (AY740401), whereas the BE424382 sequences align with the same gene at the 3′-end. 

The SBEIIb genes on the oligoarrays (Figure 6(q); Ta.4204 and Ta.11127) have a similar expression pattern to SBEIIb on the cDNA array that showed a rapid increase from 6 DPA with a peak at 14 DPA and then declined thereafter. However, the expression of SBEIIb genes as detected by the oligorray remained at higher levels after 14 DPA than did the cDNA array samples. The clone for SBEIIa (Figure 6(h), Ta.28697) detected with the oligoarray had a similar general shape to those detected by the cDNA arrays which started high at 6–8 DPA and remained high until 14 DPA after which transcript levels declined.

#### 3.2.4. Starch Debranching Enzymes

The starch debranching enzymes (DBEs) catalyze the hydrolysis *α*-1,6 glycosidic linkages important for maintaining starch granule crystallinity [2, 40]. There are two types of DBE present in plants, the pullulanase and the isoamylase. There were no DBE genes represented in the cDNA array but both were on the oligoarrays. Both pullulanase (Figure 7(b)) and isoamylase1 (Figure 7(c)) genes showed the Middle Expressor pattern with a peak at 14 DPA and gradually declined after 21 DPA. The pullulanase result is consistent with the RNA expression profile of a pullulanase gene cloned from wheat [41]. The expression of isoamylase2 (Figure 7(d)) is distinct and has not been previously reported.

### 3.3. Expression of Other Known Genes in the Sucrose to Starch Pathway

The phosphoglucose isomerase (PGI; EC 5.3.1.9) gene detected by the cDNA array encodes a plastidic isoform and was an Early Expresser (Figure 5(b)), whereas the PGI gene Ta.894 detected by the oligoarrays (Figure 6(b)), which encodes a cytoplasmic isoform, rose early in development and then remained fairly constant but with some variation in level. With triosephosphate isomerase (TPI; EC 5.3.1.1), another glycolytic enzyme, the gene detected by the cDNA array (Figure 5(r)), encoded a cytoplasmic isoform and was a Middle Expresser, similar to most of the genes encoding for starch synthesis enzymes. The TPI genes detected by the oligoarray (Figure 6(f)), included two genes for cytoplasmic isoforms, Ta.13669 and Ta.27753, and a plastidic isoform, Ta.4315. The cytoplasmic Ta.13669 was a Middle Expresser, whereas Ta.4315 and Ta.27753 show a pattern of expression that were closer to an Early Expresser profile. Phosphoglucomutase (PGM; EC 5.4.2.2) expression was detected by both the cDNA array (Figure 5(c), BF484585) and the oligoarray (Figure 6(c), Ta.24519). The PGM genes on both arrays encoded cytoplasmic isoforms of the enzyme and were Early Expressers. 

UDP-glucose pyrophosphorylase (UGPase; EC 2.7.7.9; also known as UTP-glucose-1-phosphate uridylyltransferase) is a cytosolic enzyme involved in the synthesis of starch in the developing caryopsis as part of the main path of glucose into plastids. The expression of genes encoding for UGPase expression was similar to the Early Expresser pattern as detected by both cDNA (Figure 5(a)) and oligoarrays (Figure 6(a)). 

Sucrose synthase (SuSy; UDP-glucose: D-fructose 2-glucosyltransferase, EC 2.4.1.13) catalyzes the reversible conversion of sucrose and UDP into UDP-glucose and fructose. At least three isoforms are known for maize [42] and six isoforms for Arabidopsis [43]. Two classes of sucrose synthase genes were represented on the cDNA array, with SuSy1 and SuSy2 both showing a bell-shape patterns of expression (Figures 5(i) and 5(j)). Once again there were differences in profile shape for 3′ and 5′ sequences. For the SuSy2 gene, two 3′ sequences had almost identical patterns (BE398649 and BE423294), while two 5′ sequences had either a similar, but depressed pattern compared to the 3′ sequences (BE604913), or an almost flat pattern (BE498731). The expression of the SuSy2 gene detected by oligoarrays (Figure 6(a)) gave a similar pattern as the 3′ sequences observed for the cDNA arrays but represents a different gene (by sequence analysis). The bell-shape expression pattern detected by the cDNA arrays for the Susy1 gene (Figure 5(i)) is similar to SuSy2. The SuSy1 probesets on the oligoarray (Figure 6(i)) do not distinguish the SuSy isoforms and may hybridize with other related genes.

Sucrose phosphate synthase (SPS; EC 2.4.1.14) catalyzes the conversion of fructose-6-phosphate and UDP-glucose into sucrose-6-phosphate and UDP. Recent reports have shown that plants have multiple forms of SPS and are encoded by five different families of genes in wheat [44]. The two SPS cDNA clones on the cDNA array (Figure 5(t)) represent the same SPS gene and showed modulated pattern of expression. The three SPS probesets on the oligoarrays (Figure 6(t)) showed three different patterns of expression compared to the SPS gene represented on the cDNA array. Analysis of the SPS target DNA sequences from which the probesets were designed indicated that the three SPS probes on the oligoarray and the one on the cDNA array represent four distinct genes. 

When focusing on specific known pathways, the most striking example of correlations using the cDNA arrays was for sugar accumulation and synchronous expression of glycolytic and OPPP (oxidative pentose phosphate pathway) genes (Supplementary Material 1). These pathways provide reducing power (NADH), intermediates, and ATP for starch biosynthesis. Most of these genes are early expressers; for example, FBPA (fructose-1,6-bisphosphate aldolase; Figure 5(e)), enolase (Figure 5(f)), and those not shown in Figure 5 such as a putative genes for 6-phosphogluconolactonase, cytosolic 6-phosphogluconate dehydrogenase, and transketolase.

Our results showed that most of the genes involved in starch metabolism were Middle Expressers. Genes shared between glycolysis and starch biosynthesis, however, were in general Early Expressers, suggesting that they may be at a key juncture in energy generation and utilization in a major metabolic pathway in developing endosperm. These results agree broadly with that published by Sreenivasulu et al. [45] in developing barley caryopsis where they showed that genes involved in energy production are expressed in general earlier than genes involved primarily in starch metabolism.

### 3.4. Identification of Coexpressed Regulatory Genes

Posttranslational regulation including phosphorylation, interaction with 14-3-3 regulatory proteins and posttranslational red-ox activation, appear to be essential regulatory mechanisms controlling starch biosynthesis [46] by providing a rapid response to short-term environmental changes. There are fewer examples of coarse control of starch biosynthesis, either because this is not as important or has not been as extensively studied [20, 47]. We performed correlative analysis to find genes which show synchronous expression with genes encoding proteins that regulate starch accumulation; that is, 14-3-3, PDK, WRKY TFs, and protein kinases to identify possible targets of their action.

#### 3.4.1. The 14-3-3-Proteins

**Figure 8:**
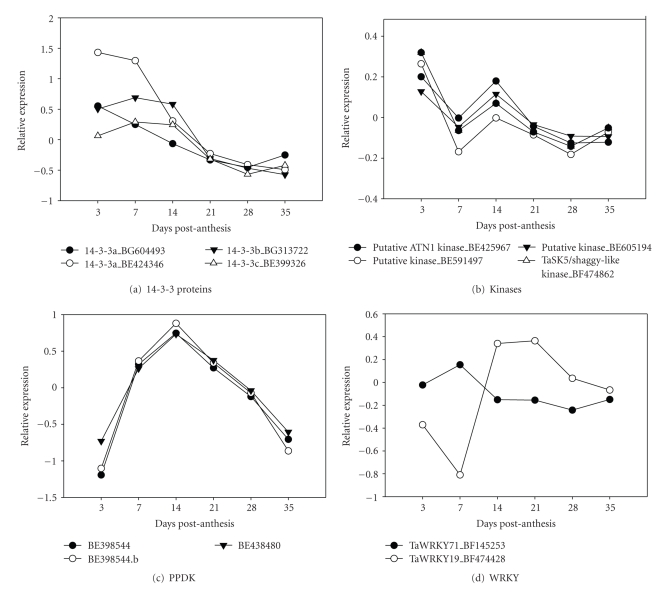
*Transcript profiles of regulatory genes. *The *x*-axis shows the developmental stage. The *y*-axis represents the relative gene expression. Expression values are given as log 2-transformed normalized relative signal intensities, so that one unit on the *y*-axis represent an expression ratio of a factor of 2. GenBank accession numbers of the clones surveyed on the array are displayed on the chart. Transcript expression profiles of (a) 14-3-3 proteins; (b) kinases; (c) pyruvate orthophosphate dikinase; (d) WRKY TFs.

These proteins enable phosphorylation and hence regulation of target proteins in eukaryotes. There are hundreds of closely related forms [48] and at least one member, a 14-3-3 protein from the *ε*-group, may directly regulate the synthesis of starch by binding SSIII [49]. In barley caryopses, as many as 16 proteins involved in carbohydrate metabolism were able to bind 14-3-3a proteins in vitro and are all putative candidates for regulation by phosphorylation in vivo [50]. Our data showed that the genes for 14-3-3b and 14-3-3c had a pattern of expression (Figure 8(a)) similar to some of the genes encoding glycolytic genes, as well as to unknown and hypothetical proteins, making it possible that they have a role in interacting with these regulatory proteins (see Supplementary Material 4).

#### 3.4.2. Protein Kinases

In plants, protein phosphorylation has been implicated in many aspects of cellular regulation and metabolism including the regulation of carbohydrate metabolism [51, 52]. We observed coordinated transcript accumulation between genes encoding putative protein kinases, a GSK-3/shaggy-related protein kinase, and a putative ATN1 kinase (Figure 8(b)). Expression pattern of these kinase genes correlated with the expression of genes coding for AATP and SPS as detected by the cDNA array and the rate of starch accumulation (see Supplementary Material 4). This similar pattern of expression may suggest that AATP and SPS are possible targets for the action of these protein kinases. There is good biochemical evidence for the regulation of starch metabolism [53] and especially of SPS [54] by protein kinases. There is now direct evidence that a gene homologue of a GSK3-like kinase called MsK4, from *Medicago sativa,* regulates carbohydrate metabolism under environmental stress [52].

#### 3.4.3. Pyruvate Orthophosphate Dikinase (PPDK)

A reduction in the expression of PPDK in rice is responsible in part for the floury-4 endosperm phenotype characterized by loosely packed starch granules and reduced starch biosynthesis in the immature endosperm [55]. PPDK expression in (Figure 8(c)) was similar to two clones of SuSy2, SBEIIb, a probable starch synthase precursor, and glycosyltransferase, as well as with several unknown proteins (see Supplementary Material 4).

#### 3.4.4. Transcription Factors

WRKY proteins are a superfamily of regulators that control diverse cellular processes. There were two clones on the array which belong to Group 3 of the WRKY superfamily [56]. The WRKY 71 clone had maximal expression at 7 DPA (Figure 8(d)), while the WRKY19a clone on the array changed sharply in expression level during the caryopsis early grain-filling stage and was synchronously expressed with two clones similar to the oat storage protein avenin, a wheat monomeric alpha-amylase inhibitor, the wheat CM2 protein, and an unknown protein (see Supplementary Material 4).

### 3.5. cDNA Array and Oligoarrays

The expression profiles of the genes involved in sucrose to starch pathway as detected by cDNA and oligoarrays showed good agreement, particularly for those genes whose isoforms were represented on both array platforms. The agreement was in spite of the fact that the two global profiling experiments use different array platforms and RNA targets from plant materials of distinct genotypes, these results differ from the report of Poole et al. [5, 7] who concluded that measures of global expression with cDNA and oligoarray experiments were not consistent after comparing relative expression levels between two cultivars at a single developmental time point [57]. A contributing factor for the discrepancy is that Poole et. al. compared the expression of genes from the cDNA and oligoarrays that are similar at the expectation value of e^−20^ or less, which could include members of gene families that have different patterns of expression. Our report focused on changes in the patterns of expression of single genes over caryopsis development within a single germplasm. The resulting profile patterns are consistent at least at a level indicating major changes in patterns. 

As mentioned, the set of genes represented in the cDNA array complemented the set in the oligoarrays and vice versa. For example, the plastidic PGI gene was present in the cDNA array, whereas the cytosolic PGI was present in the oligoarray. The cDNA array probes were sensitive enough to detect the expression of the genes coding for the different isoforms of the same enzyme. These differences in expression pattern, however, were more clearly distinguished with the isoforms detected in the oligoarrays. Although the oligoarray contains a better representation of genes, extra effort should be taken to verify the annotations for the probe sets used since a significant number of the NCBI Unigene assemblies used in the original design of short oligomers for probes have been removed and the ESTs belonging to the original assembly may now be assigned to different gene assemblies. 

Thus far, the probesets on all wheat DNA arrays available contain an incomplete representation of the transcriptome. Future arrays should take advantage of increasing sequence resources and prior array studies to develop more complete transcriptome representation. Since a complete sequence of wheat will not be available soon, it may be possible to utilize the recently complete sequence of the monocot *Brachypodium*, a closer relative of wheat than rice [58], to fill gaps in knowledge of wheat genes.

## 4. Concluding Remarks

In this study, we investigated the transcriptional control of storage starch synthesis in developing wheat caryopsis. We overlaid the results of a global gene expression profiling experiment with the analysis of soluble sugar accumulation, starch content, and starch granule particle size distribution on the same batch of biomaterials used for the microarray experiment. The 8 K cDNA array [21] used in this study was supplemented with data from an oligoarray report [22]. This approach allowed us to gain an overview of the changes in the expression of genes coding for key enzymes involved in starch biosynthesis. Genes previously not associated with starch metabolism, including those for transcription factors, were tentatively identified by which expression profile showed a positive correlation with changes in the physicochemical properties and composition of starch during caryopsis development.

### 4.1. Carbon Partitioning in Developing Caryopses

**Figure 9:**
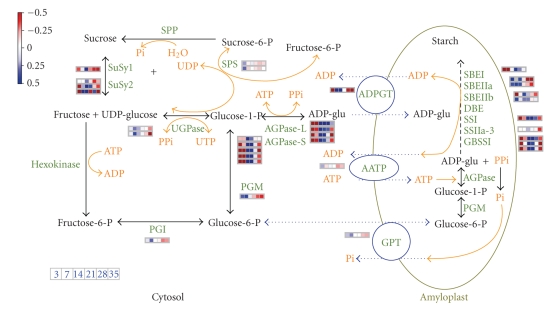
*Schematic of the metabolic flux and expression of genes involved in sucrose to starch pathway in developing wheat seed. * Black arrows indicate enzymatic reactions. Blue broken arrows represent transport of metabolites across amyloplast membranes. Enzyme and transporter names are in green. Upper box legend indicates level of gene expression and lower box legend indicates the time or developmental stage as days postanthesis. Only the expression of genes represented on the cDNA array is shown.

There are two major routes to make ADP-glucose available for the biosynthesis of storage starch in the cereal amyloplast [59]. One route is through the cytosolic synthesis of ADP-glucose (ADPG) and its transport into the plastid by the ADPG transporter; the second route involves the cytosolic synthesis of glucose-6-phosphate and its transport to the plastid by the glucose-6-phosphate/phosphate transporter (GPT). It has been suggested that the cytosolic generation of ADPG provides a more efficient way of “committing” carbon to starch biosynthesis, rather than importing hexose-phosphates, which can be used for other competing metabolic pathways in the plastid [60]. As summarized in Figure 9, our data support the idea that the major route to starch synthesis is through the cytosolic AGPase-generated ADP-glucose. UGPase gene expression remained high from 7 DPA to 28 DPA when major starch synthesis occurs. The expression of cytosolic PGM gene peaked at 7 DPA and rapidly declined thereafter. This suggests that the PGM-mediated conversion of glucose-1-phosphate to glucose-6-phosphate for subsequent export to the amyloplast plays a lesser part during maximal starch synthesis. Furthermore, the expression of the genes that encode for the cytosolic isoforms of AGPase SSU and LSU is expressed at a much higher level than the genes that encode specifically for the plastidic isoforms. Finally, the expression profile of the gene coding for the plastid transporter ADPGT expression peaked at 14 DPA when grain filling is maximal, whereas AATP and GPT expressions were downregulated suggesting their reduced role during this stage.

### 4.2. Correlative Gene Expression Analysis

Gene-to-metabolite correlations have been successfully used to provide new insights into the regulatory processes of metabolite accumulation; that is, see [61–63]. This approach was used to broaden the potential gene candidates that may regulate starch biosynthesis in wheat. However, one should be cautious when analyzing gene-metabolite correlations due to the time lag between gene expression and metabolite accumulation/phenotype observation. In our study, transcript levels were assessed at 7-day intervals, which most likely would have been long enough to compensate for the lag between transcript and metabolite accumulation/phenotype observation. It should be noted that we would have missed positive gene-to-metabolite correlations for genes that changed their expression pattern exactly at the time-points sampled.

Correlative analysis of transcript and biochemical data can be useful in identifying previously unrecognized relationships. We observed a tight correlation between the expression pattern of AATP, an SPS, several kinases, including putative kinases, and the rate of starch accumulation. One of these kinases (GSK-3) has been shown to be definitively involved in the regulation of starch metabolism [52]. Therefore the coexpression of the putative kinases with AATP, SPS and the rate of starch accumulation may prove useful for identifying regulators of starch biosynthesis, which have not yet been implicated as having a role in this process. 

Some of the starch biochemical determinants we assayed correlated very well with transcript levels of major seed storage proteins. For example, changes in starch amount, B-granule number, and volume throughout development correlated with the expression pattern of genes for low and high-molecular weight glutenins, as well as with different classes of gliadins (see Supplementary Material 1). Several studies in maize [64, 65] similarly reported a positive correlation between the abundance of the *α*-zein transcript, which encodes for a maize storage protein, and mature kernel dry weight, suggesting that *α*-zein synthesis might in some way be linked to carbohydrate formation. In wheat, genes for HMW-glutenin subunits and *AgpL* shared common regulatory loci that mapped to different chromosome arms [66]. Several mutants selected for high grain protein often have pleiotropic effects on starch, for example, high lysine barley [67, 68] and rice mutants [69]. Our data add to the long-held view that there is a global mechanism that controls the accumulation of the major storage reserves in storage organs and that sugars are the primary control elements in this process [65, 70–72]. Thus, sugars could provide an overarching mechanism to modulate storage product accumulation.

The Supplementary Materials reported in this paper are available at http://wheat.pw.usda.gov/pubs/2009/Laudencia/.

## Figures and Tables

**Table 1:** Transcripts and physiological/biochemical parameters, which showed similar correlative patterns with the change in the amount or rate of accumulation of sugars, amylose, amylopectin, and A- and B-granule. The expression patterns of genes in developing caryopses of spring wheat *T. aestivum* cv. Bobwhite were examined using RNA from six time-points with 3 biological replicates for each time-point. The gene expression patterns that correlated with the accumulation profiles of the physiological/biochemical parameters were identified using the Pearson Correlation module in GeneSpring GX Software (Santa Clara, CA). Only genes with Pearson's correlation coefficient (*r*-value) > 0.95 are shown. The Gene ID and closest BLAST N hit description (cut-off *E*-value ≤ 10^−10^; DNA database release 144) of each transcript are indicated. NA denotes not applicable. Species abbreviations—Os: *Orzya sativa*, Ta: *Triticum aestivum*; Tt: *Triticum turgidum*; *Aegilops markgrafii* (Am); At: *Arabidopsis thaliana*; Hv: *Hordeum vulgare*; Bo: *Bambusa oldhamii*.

Gene name (Species)	GenBank ID
(A)* Sugars *	

*Sucrose (mg/gDW)*	
putative transcription factor BTF3 (Os)	BG263006
sucrose:fructan 6-fructosyltransferase (Ta)	BQ806964
ATP/ADP carrier protein (Tt)	BE426214
putative cytosolic 6-phosphogluconate dehydrogenase (Zm)	BE499049
putative transcription factor (Os)	BE422879

*Fructose (mg/gDW) (top 10 genes)*	
OsCDPK protein (Os)	BE424694
Phosphoglucomutase (Ta)	BF484585
Vacuolar invertase1 (Ta)	BE470578
Putative aldose reductase (Os)	BE426211
Putative myb protein (Os)	BM134356
0.19 dimeric alpha-amylase inhibitor (Am)	BE425004
Kelch repeat-containing F-box family protein-like (Os)	BQ805590
Putative DNA methyltransferase DMT106 (Os)	BG274989
Putative UDP-glucose dehydrogenase (Os)	BQ804766
Transmembrane protein kinase (Os)	BG604519

*Glucose (mg/gDW) (top 10 genes)*	
Calmodulin (Zm)	BE495028
14-3-3 protein homologue (Hv)	BE424346
Putative transcription factor (Os)	BE404468
Cold shock protein-1 (Ta)	BF146102
Phosphoglucomutase (Ta)	BF484585
Putative transcription factor PCF3 (Os)	BQ838703
Auxin responsive protein-like (Os)	BF429053
Putative UDP-glucose dehydrogenase (Os)	BQ804766
Putative phosphogluconate dehydrogenase (Os)	BQ806618
Putative aldose reductase (Os)	BE426211

(A)* Amylose and Amylopectin *	

*Percentage Amylose*	
Amylose amount (mg/gDW)	NA
Alpha-gliadin (Ta)	BE399594
Fresh weight per caryopsis	NA
B-granule % volume	NA
Gamma-gliadin (Ta)	BE422921
Amylose rate (mg caryopsis/day)	NA
Amylose amount (mg/caryopsis)	NA
Gamma-gliadin (Ta)	BE422980

*Percentage Amylopectin (top 10 genes)*	
Auxin-induced protein (Saccharum hybrid cultivar)	BE444720
Putative zinc finger protein (Os)	BG604730
Triose phosphate translocator (Ta)	BE488921
Putative OsLRK1(receptor-type protein kinase) (Os)	BG607784
Putative pyruvate dehydrogenase E1 beta subunit (Os)	BE494481
Putative alkaline/neutral invertase (Os)	BQ806650
Putative zinc finger protein (Os)	BF201579
MADS box transcription factor (Ta)	BF429033
Thioredoxin H (Ta)	BE423204
Putative pyruvate dehydrogenase E1 beta subunit (Os)	BE399382

*Amylose rate (mg/gDW/day)*	
Sucrose synthase (Type I) (Hv)	BF474552
Brittle-1 protein; chloroplast precursor	BE399058
Sucrose synthase 2 (Bo)	BE498731
BRI1-KD interacting protein 103 (Os)	BE424602
DnaJ domain family (At)	BE497111
Nuclear inhibitor of PP1-like (Os)	BE637971

(B)* Starch granules *	

*A-granule number % (top 10 genes)*	
Putative TATA box-binding protein associated factor 10 (Os)	BE399264
Putative DEAD BOX RNA helicase (Os)	BE404899
MADS box transcription factor (Ta)	BF429033
Adenosine kinase (Zm)	BE424366
Glucose-6-phosphate dehydrogenase (Ta)	BF291587
Granule-bound starch synthase GBSSII	BE497955
MADS-box protein 9 (Hv)	BQ804479
TaWIN1 (Ta)	BE424197
Putative transcription factor (Os)	BE422879
Putative transcription factor (Os)	BE423371

*B-granule number % (top 10 genes)*	
Gamma-gliadin (Ta)	BE423485
B-granule volume %	NA
Gamma-gliadin (Ta)	BE422921
Aldolase (Os)	BE488825
B/A-granule number ratio	NA
Beta-amylase 1 (Hv)	BE423462
Water content (mg/caryopsis)	NA
Low molecular weight glutenin (Ta)	BE424477
Gamma-gliadin (Ta)	BE422980
Seed storage protein (Ta)	BQ805009

*A-granule volume %*	
Putative SNF2 domain-containing protein (Os)	BE399473
Putative zinc finger protein (Os)	BF201579

*B-granule volume % (top 10 genes)*	
Gamma-gliadin (Ta)	BE423485
B-granule number %	NA
B/A-granule number ratio	NA
Gamma-gliadin (Ta)	BE422921
Aldolase (Os)	BE488825
B/A-granule volume ratio	NA
Beta-amylase 1 (Hv)	BG262481
Triticin precursor (Ta)	BF428943
Gamma-gliadin (Ta)	BE422980
Globulin (Tt)	BE398314

**Table 2:** Transcripts with similar correlative patterns to that of different biochemical parameters were identified using Pearson Correlation module in Genespring GX Software. Only genes with Pearson's correlation coefficient (*r*-value) > 0.95 are shown. The Gene ID and closest BLAST N hit (cut-off *E*-value ≤ 10^−10^; DNA database release 144) of each transcript are indicated. Species abbreviations—Os: *Orzya sativa*, Ta: *Triticum aestivum*; At: *Arabidopsis thaliana*; Hv: *Hordeum vulgare*; Sc: *Secale cereale*; Tt: *Triticum turgidum* subs *durum*; Tp: *Thinopyrum ponticum*; Zm: *Zea mays*.

Gene Name (Species)	GenBank ID
*Sucrose (mg/gDW) and A-granule number*	

Adenosine kinase (Zm)	BE424366
SOH1-like protein (Os)	BE398213
Putative TF (Os)	BE422879
Putative plastid protein (Os)	BE423141
Sucrose:fructan 6-fructosyltransferase (Ta)	BQ806964
Putative cytosolic 6-phosphogluconate dehydrogenase (Zm)	BE499049
Putative transcription factor BTF3 (Os)	BG263006

*Glucose (mg/gDW) and A-granule number*	
14-3-3 protein homologue (Os)	BE424346
Putative 6-phosphogluconolactonase (Os)	BE443954
Putative transcription factor PCF3 (Os)	BQ838703
Putative phosphogluconate dehydrogenase (Os)	BQ806618
Putative transketolase 1 (Os)	BE422449
Putative yabby protein (Os)	BE606469
MADS-box protein 9 (Hv)	BQ804479
Putative plastid protein (Os)	BE423141
Putative transcription factor (Os)	BE423371
Putative protein kinase (Os)	BQ805311
Sucrose:fructan 6-fructosyltransferase (Ta)	BQ806964
6-phosphogluconate dehydrogenase isoenzyme B (Zm)	BF429350
SOH1-like protein (Os)	BE398213
Probable protein kinase [imported] (At)	BE424764
Putative pyruvate kinase isozyme A; chloroplast prec. (Os)	BF483010

*Fructose (mg/gDW) and A-granule number*	
Putative transcription factor PCF3 (Os)	BQ838703
Glucose-6-phosphate/phosphate translocator (Ta)	BE443396
Putative transketolase 1 (Os)	BE422449
14-3-3 protein homologue (Hv)	BE424346
Putative yabby protein (Os)	BE606469
PISTILLATA-like MADS box protein (Ta)	BQ806979
Probable protein kinase [imported] (At)	BE424764
Putative 6-phosphogluconolactonase (Os)	BE443954
Putative pyrophosphate fructose 6-phosphate 1-phosphotransferase alpha subunit (Os)	BE403152
Putative pyruvate kinase isozyme A; chloroplast prec. (Os)	BF483010

*Starch (mg/caryopsis) and B-granule number*	
Putative general negative regulator of transcription (Os)	BE500311
Putative WD-40 repeat protein (Os)	BE398510
Alpha-gliadin (Ta)	BE399594
Alpha/beta-gliadin precursor (A-III)—wheat	BE422727
Amylose rate (mg·caryopsis^−1^·day^−1^)	NA
Alpha-d-maltose; beta-amylase (Sc)	BE606197
Putative small zinc finger-related protein (Os)	BE590490
Beta-amylase (Hv)	BE423446
Putative WD domain containing protein (Os)	BE424822
Alpha-gliadin	BE422742
Beta-amylase (Hv)	BE422952
S-type low molecular weight glutenin L4-292 (Ta)	BE424629
Alpha-gliadin (Ta)	BE399836
Low molecular weight glutenin subunit (Tp × Ta)	BE423321
Alpha-gliadin (Ta)	BE399237
Alpha-gliadin storage protein	BE438304
Alpha-gliadin (Ta)	BE423477
Alpha/beta-gliadin (Ta)	BQ804641

*A- and B-granule total volume (* *μ* *m^3^)*	
B-granule volume %	NA
Gamma gliadin (Ta)	BE422921
Gamma gliadin (Ta)	BE423485
B-granule number %	NA
Gamma gliadin (Ta)	BE422980
Aldolase (Os)	BE488825
Alpha gliadin (Ta)	BE399836
Gamma gliadin (Ta)	BE399706
Alpha/beta gliadin (Ta)	BQ804641
Triticin precursor (Ta)	BF428943
Low molecular weight glutenin (Ta)	BE423268
Beta-amylase (Ta)	BE423446
Beta-amylase 1 (Ta)	BG262481
Globulin (Tt)	BE398314

## Abbreviations

UGPase: UDPglucose pyrophosphorylase
PGI: Phosphoglucoisomerase
PGM: phosphoglucomutase
GPT: glucose phosphate transporter
FbPA: fructose bisphosphatase
GBSS: Granule Bound Starch Synthase
SBE: Starch Branching Enzyme
SuSy: Sucrose synthase
AGPase: ADPglucose pyrophosphorylase (large and small subunits)
ADPGT: ADP-Glucose transporter
SS: Starch synthase
TPI: Triosephosphate isomerase
SPS: sucrose phosphate synthase
AATP: ADP/ATP Transporter
DBE: starch de-branching enzyme
TF: Transcription factor
DPA: Days PostAnthesis
NCBI: National Center for Biotechnology Information.
